# Arthroscopic Meniscectomy vs Meniscal Repair: Comparison of Clinical Outcomes

**DOI:** 10.7759/cureus.44122

**Published:** 2023-08-25

**Authors:** James Bottomley, Oday Al-Dadah

**Affiliations:** 1 Trauma and Orthopedic Surgery, South Tyneside District Hospital, South Shields, GBR; 2 Orthopedics, Faculty of Medical Sciences, Translational and Clinical Research Institute, Newcastle University, Newcastle upon Tyne, GBR

**Keywords:** patient reported outcome measures, clinical outcomes, meniscal repair, meniscectomy, meniscal tear, meniscus

## Abstract

Background

Meniscal tears are the most common injury of the knee. Surgical treatment has fallen into contention recently and includes arthroscopic meniscectomy and meniscal repair. The primary aim of this study was to quantitatively evaluate patients with isolated meniscal tears and compare their outcomes with patients who have undergone arthroscopic meniscus surgery. The secondary aim of this study was to compare the clinical outcomes of patients who have undergone arthroscopic meniscectomy with patients who have undergone arthroscopic meniscal repair.

Methods

This comparative clinical study screened 334 patients to identify subjects who underwent arthroscopic knee surgery for isolated meniscal tears and compare them to patients with symptomatic isolated meniscal tears awaiting surgery using validated patient-reported outcome measures. These included the Knee Injury and Osteoarthritis Outcome Score, International Knee Documentation Committee Subjective Knee Form, Lysholm score, Tegner score, EuroQol-5 Dimension, and the 12-Item Short Form Health Survey.

Results

A total of 117 patients (Meniscal Tear group (n=36), Meniscectomy group (n=64), and Meniscal Repair group (n=17)) were included in the final data analysis. Both the Meniscectomy group and the Meniscal Repair group (mean 55-month follow-up) showed significantly better clinical outcomes than patients in the Meniscal Tear group (p<0.05). Overall, the Meniscal Repair group demonstrated superior clinical outcomes when compared to the Meniscectomy group (p<0.05).

Conclusion

Arthroscopic knee surgery showed significant clinical benefit at medium-term follow-up in treating patients with isolated meniscal tears. When feasible, meniscal repair should be performed preferentially over meniscectomy.

## Introduction

The menisci are crescentic wedge-shaped structures formed from fibrocartilage located within the medial and lateral compartments of the knee between the corresponding femoral condyle and tibial plateau [1,2]. Meniscal tears are the most common injury to the knee joint, affecting 66/100,000 people per year [1], usually occurring during twisting activities, and can be divided into degenerative and traumatic etiology [2,3]. Approximately 80% of meniscal tears are seen in men [1], and present with a wide variety of symptoms and signs including pain, locking, catching, clicking, intermittent swelling, joint-line tenderness, or tender palpable meniscal tissue.

Treatment for meniscal tears broadly falls into two categories: conservative vs. surgical, the latter of which includes arthroscopic meniscectomy and arthroscopic meniscal repair. The choice depends on many factors including age, type and severity of tear, the presence of other pathology, and general patient fitness for anesthesia and surgery [1,3,4]. When possible, conservative treatment should be the first line, which can include analgesics, physiotherapy, and steroid injections [1,4]. Escalation to surgery can follow if symptoms do not resolve.

In general, the treatment for traumatic meniscus tears differs from that of degenerative tears. The latter are less likely to be amenable to repair as by their very definition their blood supply is poor [1,2]. Furthermore, arthroscopy is not indicated for degenerative meniscus tears in the presence of advanced arthritis as the results are poor and there is a high subsequent conversion rate to joint replacement soon after the initial arthroscopic procedure.

Surgical management includes arthroscopic meniscectomy and meniscal repair. An arthroscopic partial meniscectomy (APM), in which only the torn section of the meniscus is removed, is now the most frequently performed orthopedic operation in the United States [5].

As compared to meniscectomy, meniscal repair is a more biologically preserving procedure as it retains the native meniscal tissue within the knee joint. Multiple repair techniques have been described in the literature, the most common of which include all-inside, inside-out, and outside-in, with no significant differences in failure rates, complication rates, or clinical outcomes [6]. Outcomes are generally related to vascular supply, with superior outcomes expected in red-red and red-white zones and if surgery is performed soon after onset; poorer outcomes are linked with increasing age and smoking [7,8].

The decision to proceed with either meniscectomy or meniscal repair involves evaluating both meniscus tear characteristics (anatomic location of tear within the meniscus, reducibility of displaced meniscus tear, duration of symptoms/chronicity of injury) and patient factors (age, surgical fitness, compliance with post-operative rehabilitation). Meniscal repair has been shown to decrease the incidence of early chondral degeneration and better preserve the knee’s biomechanical properties as compared to meniscectomy [9]. Both procedures have been shown to improve clinical outcomes, with contention over the duration of benefit, with reports that by 18 months, the degree of benefit decreases further in meniscectomy than meniscal repair [10].

Meniscal repairs are more commonly performed in younger patients and have shown superior outcomes over meniscectomy in patients under 45 years of age [6]. Increased age is usually an indication for proceeding with meniscectomy over repair due to decreased vascularity of the meniscus and increased degenerative changes in the knee. However, a study by Engler et al. [5], focusing on patients over 40 years old, showed similar outcomes between meniscectomy and repair at five-year follow-up, signifying age alone should not be a contra-indication to meniscal repair.

There are a limited number of studies directly comparing clinical outcomes between meniscectomy and meniscal repair, with those identified only having short-term follow-up periods, meniscal repairs performed concomitant with anterior cruciate ligament (ACL) reconstruction, and a limited variety of patient-reported outcome measures (PROMs) included in the clinical evaluation.

PROMs quantify clinical symptomatology as directly projected by the patients themselves. Varying from general health to disease-specific, PROMs aid clinical decision-making, inform health policy strategies, and develop and refine patient-centered care [11]. Generic PROMs provide an overview of health-related quality of life across the population, encompassing multiple diseases, but lack sensitivity to a singular etiology. Disease-specific PROMs focus on characteristics commonly seen in the condition of interest, increasing their sensitivity to detect change, whilst compromising the holistic view of the patient’s health [12]. It is therefore recommended to utilize a combination of generic and disease-specific measures.

The primary aim of this study is to quantitatively evaluate patients with isolated meniscal tears using validated PROMs and compare their outcomes with patients who have undergone arthroscopic meniscus surgery. The secondary aim of this study is to compare the clinical outcomes of patients who have undergone arthroscopic meniscectomy with patients who have undergone arthroscopic meniscal repair. The primary hypothesis is that arthroscopic surgery improves symptoms of meniscus tears. The secondary hypothesis is that arthroscopic meniscal repair has superior clinical outcomes as compared to arthroscopic meniscectomy.

## Materials and methods

This is a retrospective observational clinical study. All the patients included in this study attended a specialist knee clinic and underwent arthroscopic knee surgery following clinical assessment and radiological investigation. This study was exempt from Institutional Review Board (IRB)/Ethics Committee approval as it was a pragmatic study evaluating the existing clinical practice of the senior author (consultant orthopedic surgeon). This study was registered with the hospital’s Clinical Effectiveness Department (registration number CA9828). This therapeutic research study constituted the first author’s Masters dissertation.

This study compared the clinical outcomes of three separate groups. The Pre-Operative group included patients with isolated meniscal tears of the knee joint, the Meniscectomy group included patients who had undergone an arthroscopic meniscectomy, and the Meniscal Repair group included patients who had undergone an arthroscopic meniscal repair.

Exclusion criteria consisted of further surgery or further injury to the affected limb, any functionally limiting illness or disease, advanced knee osteoarthritis and concurrent ACL, posterior cruciate ligament (PCL), or lateral collateral ligament (LCL) tears. The presence of any of these factors could confound surgical outcomes reported. Exclusion of these cases ensured any symptoms expressed were attributable only to the original meniscal injury and their surgical treatment. Patients with medial collateral ligament (MCL) tears were not excluded as concurrent MCL tears are relatively common with meniscus tears and the former are predominantly treated conservatively and unlikely to confound the outcome of meniscus surgery.

Subjects in the Pre-Operative group constituted a cohort of patients currently on the waiting list for knee surgery who have been clinically (history and physical examination) and radiologically (magnetic resonance imaging (MRI)) diagnosed with an isolated meniscal tear and whose symptoms were refractory to conservative treatment (i.e., analgesia, physiotherapy, etc.). The Pre-Operative group allowed for a benchmark from which to compare both surgical treatment options.

Surgically treated patients in both the Meniscectomy group and the Meniscal Repair group were identified through the consultant’s surgical logbook and theatre records. All arthroscopic knee surgeries performed from August 2013 to June 2021 were reviewed using the hospital electronic health record system; MediTech version 6 (Medical Information Technology Inc., Westwood, MA, USA). The Novel Coronavirus (COVID-19) global pandemic had implications for this study as all routine elective surgical procedures (including knee arthroscopies) were canceled for an extended period of time (March 2020 onward), reducing the number of potential participants that could be recruited into this study [13,14].

All meniscal repairs included in this study were performed by using an all-inside technique using FAST-FIX 360 (Smith & Nephew plc., London, UK) for tears located at the posterior horn and middle third (body) of the meniscus. For anterior horn meniscus tears, an outside-in meniscal repair technique was performed using a 1 PDS II (polydioxanone) violet suture (Ethicon, Johnson and Johnson Health Care Systems Inc., NJ, USA). Microfracture around the margin of the intercondylar notch was performed at the same time in order to perforate the subchondral bone and introduce mesenchymal stem cells into the knee joint which in turn optimizes the biological milieu for the meniscal tissue healing process [15]. Post-operative physiotherapy rehabilitation included full weight in a knee brace with a range of movement (ROM) restricted from 0^o^ to 90^o^ for six weeks in order to protect the meniscal repair site at the initial healing phase, thereafter the brace was discontinued, and full ROM progressed. All meniscectomies were performed using standard a basket punch followed by a motorized oscillating shaver (Smith & Nephew plc.) whereby only the torn and damaged area of meniscal tissue was resected back to a stable rim (partial meniscectomy). Post-operative physiotherapy rehabilitation included full weight and full ROM without any knee brace or functional restrictions. Patient notes were perused to ascertain the type of arthroscopic knee surgery performed, conformance with the inclusion and exclusion criteria, and documenting intra-operative findings, including meniscus tear laterality (medial/lateral/bilateral), tear configuration, and specific anatomic location (anterior horn, posterior horn, middle third/body).

Data from validated patient-reported outcome measures (PROMs) regarding meniscus-related symptomatology was collected from all three groups. This included the Knee Injury and Osteoarthritis Outcome Score (KOOS) [16,17], International Knee Documentation Committee Subjective Knee Form (IKDC) [12, 18], Lysholm score [19], Tegner score [19], EuroQol-5 Dimension (EQ-5D-5L) [20], and the 12-Item Short Form Health Survey (SF-12) [21]. The PROMs for the Pre-Operative Group were completed by the patients at the time of their outpatient clinic attendance whilst in the waiting room area. The PROMs for both the Meniscectomy group and the Meniscal Repair group (and some patients in the Pre-Operative group) were obtained via posting the PROM forms to the patient’s home residence. To maximize compliance, patients were contacted by telephone before postal questionnaires were dispatched. Personalized cover sheets were also created. These provided the opportunity to obtain further information including whether patients had incurred further injury or undergone further surgery to their affected knee. Due to the extended follow-up period of up to eight years, this allowed for the appropriate exclusion of these patients as their current symptoms may not be attributable to the original meniscus surgery.

Statistical analysis

Plotted histograms with fitted curve lines, box plots, normal Q-Q plots, and the Shapiro-Wilk statistic were used to test the normality of data distribution. Almost all the PROM data (continuous variables) displayed a skewed distribution and therefore the relevant non-parametric statistical tests were used for the data analysis. The Kruskal-Wallis H test accompanied with Dunn’s post-hoc pairwise comparison test was used for the three-way group data analysis. The level of statistical significance was set at p<0.05. Statistical analysis was performed using SPSS for Windows version 26.0 (IBM Corp., Armonk, NY).

## Results

**Figure 1:**
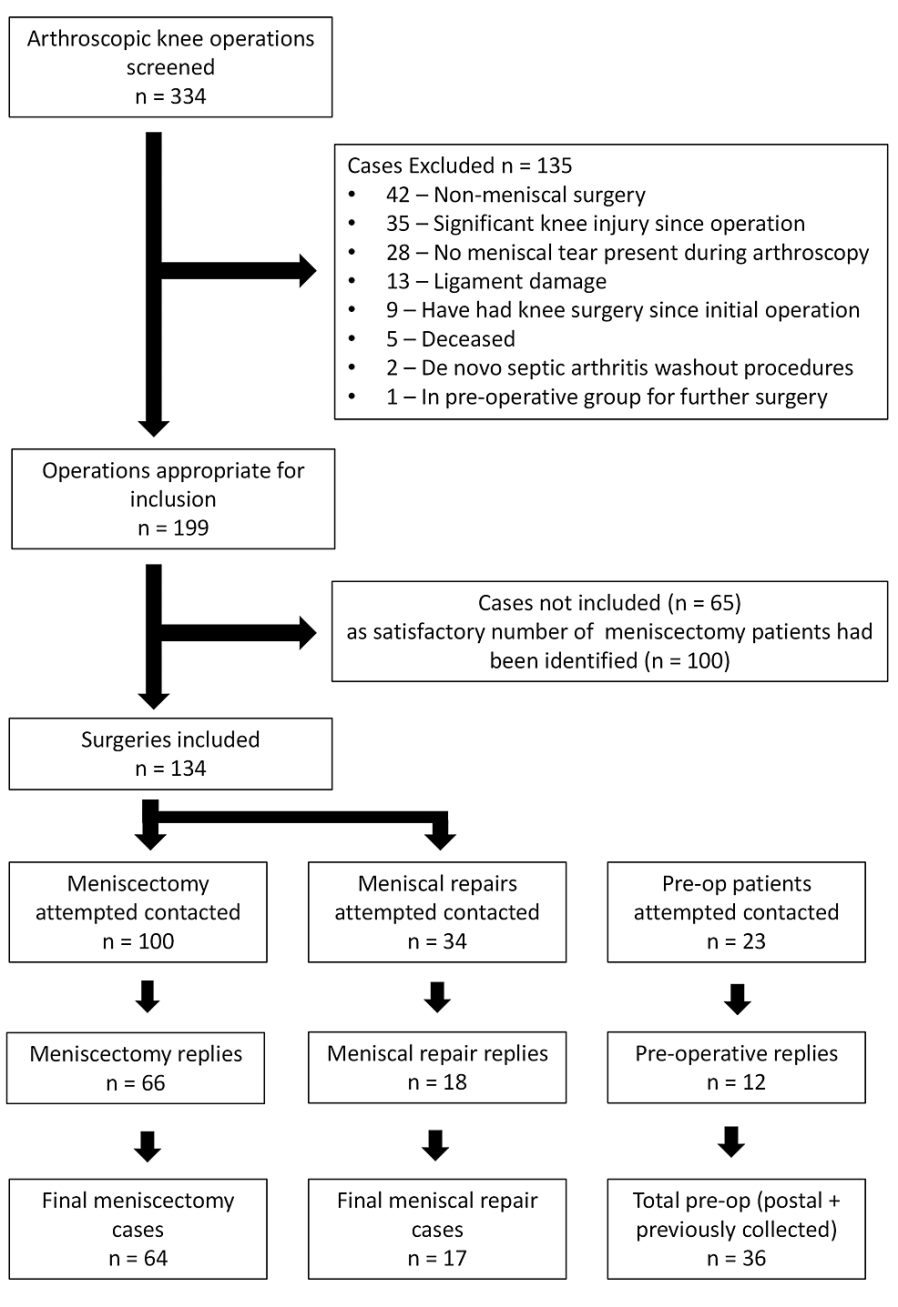
Patient flow diagram

Figure 1 shows the flow of patients through the study. A total of 334 patients from the specialist knee clinic were screened, with 157 patients contacted, resulting in 117 patients in total included in the study. Two patients were excluded from the Meniscectomy Group post reply, one due to developing osteosarcoma and another completing PROMs regarding the incorrect knee. One patient was also excluded from the Meniscal Repair group post reply due to development of septic arthritis in the joint of interest.

**Figure 2:**
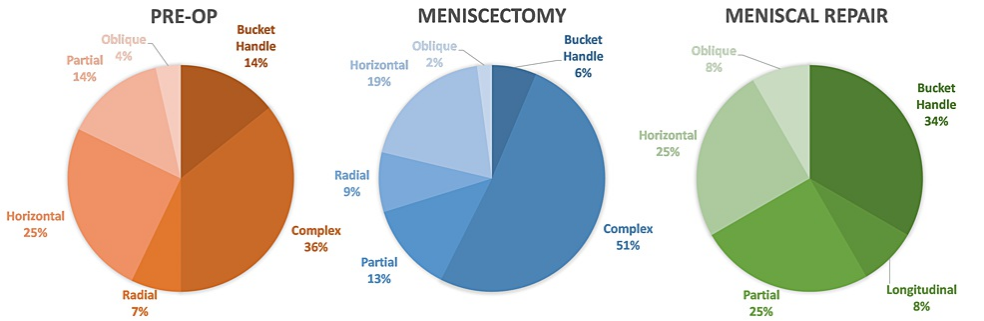
Meniscal tear pattern

Baseline demographics for all three groups are summarized in Table 1. The mean age of Meniscectomy group was older than the other two groups as the meniscal tear pattern in older patients was less likely to fit the criteria considered suitable for a meniscal repair procedure. The meniscal tear pattern and anatomical location between the groups are illustrated in Figure 2 and Table 2, respectively. The greatest proportion of complex tears was observed in the Meniscectomy cohort, whereas the Meniscal Repair group demonstrated the largest proportion of bucket handle tears. The most common location for tears across all groups was the posterior horn.

**Table 1:** Patient demographics SD: standard deviation, n/a: not applicable

	Pre-operative	Meniscectomy	Meniscal Repair
Number of patients	36	64	17
Age (mean (SD))	48 (14.5)	61 (11.3)	47 (17.7)
Gender (female : male)	9 : 27	28 : 36	4 : 13
Knee Laterality (right : left)	18 : 18	31 : 33	10 : 7
Smoking (yes : no)	6 : 29	3 : 60	2 : 15
Follow-up time from surgery (months) (mean, range)	n/a	57 (14 - 91)	47 (16 - 86)
Laterality of Meniscal Tea (medial : lateral : bilateral)	25 : 8 : 3	50 : 9 : 5	9 : 5 : 3

**Table 2:** Anatomical location of meniscal tears

Anatomical location of tear	Pre-operative, n = 36	Meniscectomy, n = 64	Meniscal repair, n = 17
Posterior Horn	16	39	10
Posterior Horn extending to Body	12	13	2
Body	6	9	2
Anterior Horn	2	3	3

Table 3 shows the between-group statistical analysis of all the PROM scores for all three groups. The results of the three-way analysis (Kruskal-Wallis H Test) showed statistically significant differences (p<0.05) between the three groups in all PROMs except for the EQ-5DVAS (p=0.103) and the mental component score (MCS) of the SF-12 (p=0.226). This implies that, overall, patients with meniscal tears clinically improved following surgery. Post-hoc analysis between the Pre-Operative group and the Meniscal Repair group showed significantly better outcomes are demonstrated across all PROMs analyzed in the latter group. A similar finding was observed in the post-hoc analysis between the Pre-Operative group and the Meniscectomy group whereby the latter demonstrated significantly greater scores in all PROMs except for the Tegner scores (both pre-injury and current) and the physical component score (PCS) of the SF-12. The post-hoc analysis between the Meniscectomy group and the Meniscal Repair group showed significantly better outcomes in the latter group in the majority of PROMs assessed.

**Table 3:** Clinical outcome analysis *Statistically Significant at p<0.05 ^1^Kruskal-Wallis H Test ^2^Dunn’s Post-hoc Pairwise Comparison Test Abbreviations: Knee Injury and Osteoarthritis Outcome Score (KOOS), Activities of Daily Living (ADL), Sports and Recreation (Sports/Rec), Quality of Life (QoL), International Knee Documentation Committee Subjective Knee Form (IKDC), EuroQol-5 Dimension (EQ-5D), 12-Item Short Form Health Survey (SF-12), Not Significant (n/s).

	Pre-operative n = 36 Median (IQR)	Meniscectomy n = 64 Median (IQR)	Meniscal Repair n = 17 Median (IQR)	P-value^1^	H	Pre-operative vs Meniscectomy^2^	Pre-operative vs Meniscal Repair^2^	Meniscectomy vs Meniscal Repair^2^
KOOS								
Pain	44.0 (31.0 - 55.3)	58.0 (36.0 - 89.0)	86.0 (66.5 - 92.0)	<0.001*	20.2	0.002*	<0.001*	0.022*
Symptoms	54.0 (36.0 - 61.0)	68.0 (46.0 - 86.0)	86.0 (68.0 - 93.0)	<0.001*	18.3	0.003*	<0.001*	0.033*
ADL	46.0 (35.0 - 70.5)	65.5 (37.3 - 87.3)	90.0 (78.0 - 96.5)	<0.001*	17.4	0.039*	<0.001*	0.003*
Sports/Rec	25.0 (5.0 - 36.3)	35.0 (15.0 - 70.0)	77.5 (66.3 - 85.0)	<0.001*	17.1	0.023*	<0.001*	0.007*
QoL	25.0 (13.0 - 31.0)	44.0 (19.0 - 69.0)	56.0 (50.0 - 69.0)	<0.001*	21.7	0.002*	<0.001*	0.016*
Overall	37.0 (23.2 - 48.1)	50.7 (36.8 - 80.9)	79.8 (65.8 - 82.6)	<0.001*	20.4	0.002*	<0.001*	0.029*
IKDC	35.1 (22.1 - 43.4)	43.7 (28.2 - 71.3)	66.7 (54.1 - 76.5)	<0.001*	17.8	0.008*	<0.001*	0.016*
Lysholm	47.0 (26. - 58.0)	61.5 (39.5 - 85.6)	84.0 (71.0 - 87.5)	<0.001*	23.5	0.001*	<0.001*	0.017*
Tegner								
Pre-injury	5.0 (4.0 - 9.0)	5.0 (3.0 - 7.0)	8.5 (5.0 - 10.0)	0.027*	7.2	0.173	0.009*	0.151
Current	2.0 (1.0 - 4.0)	3.0 (2.0 - 4.0)	4.5 (2.3 - 8.5)	0.011*	9.0	0.162	0.003*	0.031*
EQ-5D _Index_	0.57 (0.38 - 0.68)	0.69 (0.42 - 0.84)	0.77 (0.64 - 0.84)	0.001*	15.0	0.005*	<0.001*	0.081
EQ-5D _VAS _	70.0 (51.3 - 80.0)	70.0 (50.0 - 82.3)	80.0 (66.3 - 85.0)	0.103	4.5	n/s	n/s	n/s
SF-12								
PCS	31.1 (26.8 - 37.1)	35.6 (24.3 - 48.0)	49.5 (43.0 - 53.7)	<0.001*	19.1	0.094	<0.001*	0.001*
MCS	46.3 (40.6 - 54.0)	50.0 (39.7 - 58.2)	52.9 (45.5 - 58.8)	0.226	3.0	n/s	n/s	n/s

**Figure 3:**
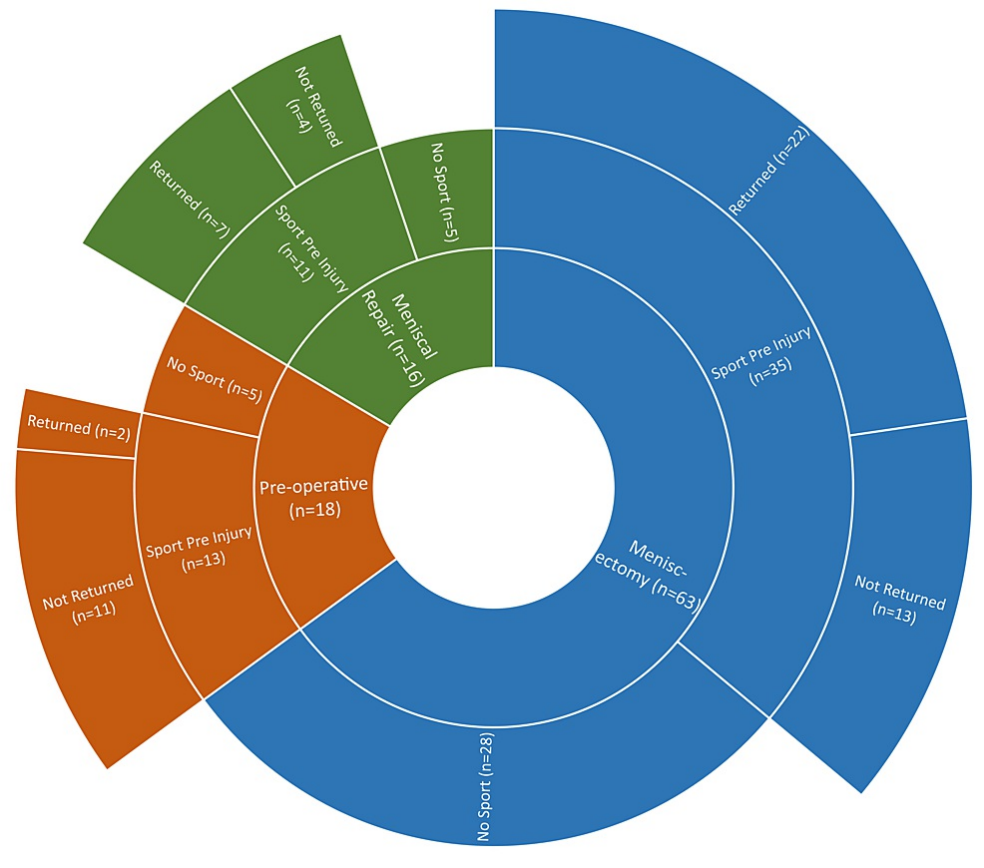
Rate of exercise: pre-injury vs current

Figure 3 illustrates the rate of exercise completion in patients pre-injury and proportion of patients which returned to exercise post-injury/intervention. As shown, the rates of exercise completion pre-injury were greater in the Pre-Operative (72%) and Meniscal Repair (69%) groups than Meniscectomy group (54%). Both operative groups had roughly 63% of active individuals pre-injury return to sport at follow-up, whereas only two out of 13 Pre-Operative patients had managed to do so (18%).

## Discussion

This study has shown that patients with isolated meniscus tears clinically improved following arthroscopic knee surgery as determined by validated PROM scores. Overall, the clinical outcome of arthroscopic meniscal repair surgery was superior to that of arthroscopic meniscectomy.

This study found significantly different outcomes between pre-operative patients with an isolated meniscal tear and those who had undergone either a meniscectomy or meniscal repair surgery. The highest scores across all PROMs were observed in the Meniscal Repair group, with the lowest scores mostly seen in the Pre-Operative group. This study is the first to quantitively evaluate pre-operative patients with isolated meniscal tears using a wide variety of validated PROMs (six in total including a combination of generic and disease-specific instruments), allowing a standardized benchmark from which to compare surgical interventions and other modalities aimed at treating meniscus pathology. The results from this study demonstrate significant clinical benefit from both meniscectomy and meniscal repair surgery at a mean 55-month follow-up, a finding also shown by a similar study by Engler et al. [5] focusing on subjects over 40 years of age. Engler et al. [5] found over 85% of patients were satisfied or very satisfied with meniscectomy or meniscal repair at five-year follow-up. The results of the present study demonstrate medium-term benefit is maintained for this follow-up period in both surgical procedures, affirming their efficacy for the treatment of meniscal tears. However, Engler et al. [5] found no differences in outcomes between meniscectomy and meniscal repair cohorts, with median IKDC scores of 78 and 77, respectively. By contrast, this study found superior outcomes in the Meniscal Repair cohort, with a median IKDC score of 43.7 following meniscectomy and 66.7 following repair (p=0.016). Greater IKDC scores could be expected in the study by Engler et al. [5] as 46.4% and 49.3% of their Meniscal Repair and Meniscectomy cohorts, respectively, had concomitant ACL reconstruction performed, which has been shown to reduce failure rates and improve clinical outcomes [8,10,22]. This is due to the release of mesenchymal stem cells into the knee joint, a consequence of drilling ACL graft tunnels within the subchondral bone of the femur and tibia, which stimulates the biological healing process of the repaired meniscus tissue. Patients with concurrent ACL reconstruction were not included in this study; therefore, the outcomes are less directly comparable with other studies. The current study offers a unique insight into the outcomes following meniscectomy and meniscal repair surgery for isolated meniscal tears.

Historically, APM has been used in a non-specific manner in patients with knee pain and any form of tear [1,22]. This generalized approach has led to contention over the validity of APM, with multiple reviews showing no benefit in either pain or function in comparison to exercise therapy or conservative management [23,24]. Clinical practice guidelines previously published have strongly recommended against arthroscopy in degenerative meniscal tears [25], arguing the potential short-term benefit does not justify increased cost and surgical risks including thromboembolism and infection. However, a critical systematic review by Hohmann et al. [26] deemed studies comparing APM and physical therapy to have a high risk of bias, low quality, and diverse study characteristics. A lack of literature comparing physical therapy to Meniscal Repairs prevents a direct comparison between these two interventions. The care of the patients included in this study was in line with the 2018 BASK meniscal surgery guidelines [4]. This includes avoidance of operative intervention in patients with advanced osteoarthritis (KL grade 4), and the implementation of non-operative treatment prior to surgery when appropriate. The adherence to these practices could explain the superior outcomes observed in the meniscectomy and meniscal repair cohort, as interventions are more targeted to those refractory to physical therapy, and where the meniscal pathology is the primary cause of symptoms. This study would therefore encourage implementation of BASK guidelines to improve patient selection and maximise the potential benefit from meniscal procedures.

The secondary hypothesis of this study was affirmed, finding significantly superior scores in the Meniscal Repair group across almost all PROMs, suggesting Meniscal Repairs have superior outcomes to Meniscectomy for isolated meniscal tears at medium-term follow-up. This finding is corroborated by a meta-analysis by Xu et al. [6], which showed greater IKDC, Lysholm, and Tegner scores following Meniscal Repair. However, the median difference in Lysholm score between meniscectomy and meniscal repair reported by Xu et al. [6] was 4.42, translating to minimal clinical benefit and is substantially less than the median difference seen in the current study of 22.5, which represents a much more profound clinically important difference in terms of patient-reported symptomatology. Greater differences could be expected in this study, as all surgical procedures (including both meniscectomy and meniscal repairs) were performed by a single specialist consultant knee surgeon, and most studies investigated in the meta-analyses were performed over 20 years ago [6,27-29]. Advancements in surgical technique and technology, particularly in meniscal repair surgery, allow this study to demonstrate the current true potential benefit of meniscal repair. The pre-injury Tegner scores were not significantly different between the Meniscectomy group and the Meniscal Repair group in this study, implying similar activity levels and functional demands before their meniscal injury. There was no difference in the EQ-5Dindex which might represent a Type II statistical error in light of the fact that all the other PROM results were significantly different and might be explained by the more generic nature of the EQ-5D as compared to the other disease-specific PROMs.

The superior outcomes observed in this study in the Meniscal Repair cohort could be attributed to preservation of the meniscal tissue and therefore retaining the three key functions they play in the knee. First, they absorb axial loading forces through the joint, by converting them into hoop stresses within the tissue. Second, the menisci stabilize the knee by improving articular congruency between the flat tibial plateaus and concave femoral condyles. Third, they modulate the gliding of the articular surfaces. The removal of the meniscal tissue by meniscectomy impacts its ability to perform such functions and could be attributed to the greater rate of secondary osteoarthritis other studies have observed in this group [30].

The menisci are innervated by the posterior articular branch of the tibial nerve. Similar to the ACL, they too contain mechanoreceptors that contribute to the proprioceptive function of the knee [31]. Studies have shown that meniscal tears result in a significant deficit in knee proprioception and despite a clinically successful APM, the proprioceptive deficit persisted following surgery [32]. A partial meniscectomy removes potentially recoverable mechanoreceptors which leave the knee persistently lacking in its proprioceptive capacity [32]. Meniscal Repairs preserve meniscal tissue (and therefore the mechanoreceptors within them) and may have a more favorable influence in this regard. The impact of proprioceptive acuity may have also contributed to the superior clinical outcomes of the Meniscal Repair group as compared to the Meniscectomy group. The proportion of complex meniscal tears in the Meniscectomy group (51%) was double that seen in Meniscal Repairs (25%). This can be expected due to decreased reducibility and complicated repair mechanisms. In contrast, bucket handle tears, a tear type more amenable to repair in which there is one primary lesion, were observed almost six times as frequently in the Meniscal Repair group (34%) as compared to the Meniscectomy cohort (6%).

A general comparison between the two operations shows that despite meniscal repair having greater day-of-surgery costs ($7094 vs $5423, respectively) [33], greater rates of 30-day complication (1.2% vs 0.84%) [33], and higher re-operation rates than meniscectomy (20.7% vs 3.9%) [22], the 10-year economic burden of meniscal repair is less than that of meniscectomy ($22,590 vs $31,528, respectively) [34]. This long-term cost could be attributed to a greater proportion of meniscectomized patients developing secondary osteoarthritis (10% vs 17%) at 10-year follow-up, subsequently requiring total knee replacements (TKR) [33,34]. This study also suggests that both meniscectomy and meniscal repair patients are three times more likely to have returned to sport at follow-up (63%) than patients who are awaiting surgery (18%). It could have been expected for a greater proportion of patients to return to sport in the Meniscal Repair cohort due to the preservation of meniscal tissue given the many key biomechanical functions it serves in the knee. 

The main limitation of this study was patient participation and recruitment. This clinical research study was conducted during the novel coronavirus (COVID-19) global pandemic, during which all routine elective surgery had ceased due to nationwide restrictions, resulting in a reduction of individuals eligible to take part. Additionally, due to the nature of postal questionnaires, patients were understandably apprehensive about receiving an envelope that had originated from a hospital during this time, further reducing the compliance rate. This study did not analyze longitudinal patient data of the two surgical groups as pre-operative data was not collected for these patients specifically. However, a suitable comparable group of pre-operative patients, refractory to conservative treatment, on a waiting list for meniscal surgery was identified and used as a benchmark from which to assess and compare surgical outcomes. The range of follow-up periods was relatively broad for both groups. However, with a mean follow-up period of 57 and 47 months for the meniscectomy and meniscal repair cohorts, respectively, this study provides a useful perspective on medium-term outcomes following meniscal surgery.

Despite limitations forced upon clinical research studies performed during the global pandemic, this analysis is based on comprehensive selection criteria and contributes key interpretation regarding the direct comparison between meniscectomy and meniscal repair in isolated meniscal tears, something only a limited number of papers have done in the past [10,35]. These findings are therefore likely to have significant implications on aiding clinical decision making when proceeding with meniscal surgery.

Suggestions for future research in this field include undertaking a multicenter study that can allow for a larger cohort of patients to be investigated, particularly in the Meniscal Repair group, whilst encompassing various meniscal surgical techniques, to provide more generalizable results. It is likely that specific patient subgroups benefit by varying amounts from each surgical procedure. Larger cohorts would also aid identification of these predictive factors, allowing adequate treatment selection. Implementation of a national database of meniscal operations, similar to the National Joint Registry and the National Hip Fracture Database in the United Kingdom, would also confer these benefits on a larger scale.

## Conclusions

This study has demonstrated a significant clinical benefit in patients with isolated meniscal tears undergoing arthroscopic knee surgery. The outcomes of meniscal repair were superior to that of meniscectomy at medium-term follow-up. Meniscal repair is a more biologically preserving procedure of the knee joint and should be performed preferentially over meniscectomy in isolated meniscal tears when feasible. The findings of this study will greatly benefit both clinicians and patients regarding the management of meniscal tears and better inform public health resource allocation.
